# Ethanol extract of *Cirsium japonicum* attenuates hepatic lipid accumulation via AMPK activation in human HepG2 cells

**DOI:** 10.3892/etm.2014.1698

**Published:** 2014-04-25

**Authors:** YUN WAN, LI-YA LIU, ZHEN-FENG HONG, JUN PENG

**Affiliations:** 1 Academy of Integrative Medicine, Fujian University of Traditional Chinese Medicine, Fuzhou, Fujian 350122, P.R. China; 2 Fujian Key Laboratory of Integrative Medicine on Geriatrics, Fujian University of Traditional Chinese Medicine, Fuzhou, Fujian 350122, P.R. China

**Keywords:** *Cirsium japonicum*, nonalcoholic fatty liver disease, AMP-activated protein kinase, fatty acid oxidation

## Abstract

One of the most common causes of chronic liver disease, nonalcoholic fatty liver disease (NAFLD), is strongly associated with obesity and dysregulated insulin action in the liver. However, there are no pharmacological agents currently established for the treatment of NAFLD. A flowering plant in the Asteraceae family, *Cirsium japonicum* (CJ), exhibits a variety of pharmacological and antioxidative properties that promote hepatoprotection. In the present study, CJ ethanol extract was shown to reduce hepatic triglyceride (TG) and cholesterol accumulation. CJ significantly increased AMP-activated protein kinase (AMPK) phosphorylation in HepG2 hepatocytes and downregulated the level of the target genes, acetyl-CoA carboxylase and fatty acid synthase. In addition, CJ upregulated the expression of carnitine palmitoyltransferase-1, which is involved in fatty acid oxidation. The results of the present study indicated that the positive effects of CJ extract on high-fat diet-induced hepatic TG accumulation were mediated via the AMPK signaling pathway, indicating a potential target for the preventative treatment of NAFLD.

## Introduction

Nonalcoholic fatty liver disease (NAFLD) is characterized by increased fat deposits in the liver and can precede more severe diseases, including nonalcoholic steatohepatitis, cirrhosis and in certain cases, hepatocellular carcinoma (1). According to a previous epidemiological survey, NAFLD is highly prevalent, affecting ~20% of the general population (2). In addition, NAFLD is strongly associated with metabolic syndrome and associated obesity, type 2 diabetes mellitus, arterial hypertension and dyslipidemia. NAFLD is considered to be the major hepatic component of metabolic syndrome. Despite large involvement in key human health crises, the pathogenesis of NAFLD is not completely understood; although the pathogenesis is considered to be multifactorial, including genetic, environmental and behavioral variables (3). As a result, current treatments for patients with fatty liver disease include lifestyle alterations and targeting the reduction of elevated lipolysis and high circulating free fatty acid (FFA) levels (4,5). Preventing increased circulating FFA levels in the liver, which can trigger a series of biological changes in hepatic lipid metabolism, may be an effective therapy for multiple stages of NAFLD (6). Cellular FFA loading is commonly utilized to develop *in vitro* models of steatosis and these models can reliably reproduce key features of hepatic steatosis in humans (7,8).

5′-AMP-activated protein kinase (AMPK) is a critical modulator of pathways involved in hepatic lipid metabolism. This kinase plays a central role in lipid metabolism regulation by activating fatty acid oxidation pathways and inhibiting lipid synthesis (7). AMPK is a heterotrimeric protein consisting of one catalytic subunit (α) and two non-catalytic subunits (β and γ). In response to an elevated cellular AMP/ATP ratio, AMPK is physiologically activated by phosphorylation at the threonine 172 (Thr172) residue in the α-subunit (9). AMPK activation is responsible for metabolic modifications (10) via the phosphorylation of downstream substrates, including acetyl-CoA carboxylase (ACC), the rate-limiting enzyme in fatty acid biosynthesis. Cholesterol production in the liver is also inhibited by AMPK activation via the suppression of HMG-CoA reductase (11). AMPK inhibits *de novo* fatty acid synthesis by inactivating ACC and promoting fatty acid oxidation by upregulating the gene expression levels of carnitine palmitoyltransferase-1 (CPT-1) and peroxisome proliferator-activated receptor (PPAR)-α (12). Fatty acid synthase (FASN) is a rate-limiting enzyme involved in fatty acid biosynthesis, catalyzing the final step in this pathway.

As an effective medical therapy for NAFLD is yet to be established, developing therapeutic agents for NAFLD is critical. Previous studies have focused on Chinese herbal remedies that can suppress hepatic lipid accumulation, including *Cirsium japonicum* (CJ) (13). The positive biological effects are wide-ranging, with CJ functioning as a cancer chemopreventive agent, a powerful anti-inflammatory and an antioxidant. Although numerous beneficial roles of CJ have been hypothesized, there have been no studies investigating the role of CJ in the regulation of genes associated with hepatocellular lipid metabolism. If CJ is hepatoprotective, understanding the genetic effects that the extract has may yield new insight into potential therapies for a variety of liver diseases. Thus, the present study investigated whether CJ can attenuate hepatic lipid accumulation through activating AMPK utilizing human HepG2 cells.

## Materials and methods

### Materials and reagents

Dulbecco’s modified Eagle’s medium (DMEM), fetal bovine serum (FBS), penicillin-streptomycin and trypsin-EDTA were purchased from Gibco-BRL (Carlsbad, CA, USA). TRIzol reagent and dimethyl sulfoxide (DMSO) were purchased from Invitrogen Life Technologies (Carlsbad, CA, USA). Oleic acid (OA) and palmitic acid (PA) were purchased from Sigma-Aldrich (St. Louis, MO, USA). Polyclonal antibodies against AMPK, phospho-AMPK, ACC, phospho-ACC, CPT-1, FASN and β-actin were obtained from Cell Signaling Technology, Inc., (Danvers, MA, USA). Assay kits for triglyceride (TG), total cholesterol (TC) and Oil Red O staining were obtained from the Jiancheng Institute of Biotechnology (Nanjing, China). Other reagents and chemicals used were of the highest grade commercially available.

### Preparation of ethanol extract from CJ

Authentic plant material was purchased from Guo Yi Tang Chinese Herbal medicine store (Fujian, China). The stock solution of CJ in the cell-based experiments was prepared by dissolving pure CJ ethanol extract in 50% DMSO and 50% phosphate-buffered saline (PBS) to a concentration of 500 mg/ml. Diluting the stock solution in cell culture medium established the working concentration. The final DMSO concentration in the medium for all the cell experiments was <0.1%.

### Cell culture and viability assay

HepG2 cells, a human hepatoma cell line, were obtained from a cell bank of the Chinese Academy of Science (Shanghai, China). Cells were cultured in DMEM supplemented with antibiotics (100 U/ml penicillin A and 100 U/ml streptomycin) and 10% heat-inactivated FBS, and were maintained at 37°C in a humidified incubator containing 5% CO_2_. Cells were subcultured at 80–90% confluence. Cell viability was determined using the 3-(4,5-dimethylthiazol-2-yl)-2,5-diphenyltetrazolium bromide (MTT) assay. In brief, HepG2 cells were seeded at a density of 3×10^4^ cells/well in a 96-well plate. The following day, the culture medium was replaced with fresh medium containing various concentrations of CJ, which was maintained for 24 h. Cells were subsequently exposed to 1 mmol/l HFFA (OA and PA at a 2:1 ratio) or equal DMSO in fresh medium for 24 h. Following treatment, 10 ml MTT (5 mg/ml in PBS) was added to each well and the samples were incubated for an additional 4 h at 37°C. The purple-blue MTT formazan precipitate was dissolved in 100 ml DMSO and the absorbance was measured at 570 nm using an ELISA reader (ELx800; BioTek Instruments, Inc., Winooski, VT, USA).

### Oil Red O staining

HepG2 cells were grown in six-well plates at a density of 7×10^4^ cells/well for 24 h. Stock solutions of 0.6 mmol/l OA and 0.6 mmol/l PA were prepared in DMSO. Following CJ and HFFA (OA:PA, 2:1) treatment for 24 h, the cells were washed twice with PBS and stained with Oil Red O for 15 min. Cell nuclei were labeled by brief exposure to hematoxylin solution, and were washed with PBS. The samples were observed under a microscope at a magnification of ×200 and ×400.

### Intracellular TG and TC measurements

To induce cellular fat-overloading, HepG2 cells at 60% confluence were cultured with or without 1.00 mmol/l HFFA (OA:PA, 2:1) in the presence or absence of CJ at 0.5 or 1 mg/ml. After 24 h, the cells were washed twice with 1 ml cold PBS. Intracellular TG and TC levels were measured in the HepG2 cell lysates and the concentrations were determined using commercial kits based on phosphoglycerol oxidase/peroxidase enzymatic reactions, according to the manufacturer’s instructions.

### RNA extraction and quantitative polymerase chain reaction (PCR) analysis

Total RNA was extracted using TRIzol reagent, according to the manufacturer’s instructions. To obtain cDNA, 1 μg total RNA was reverse-transcribed using PrimeScript^®^ II 1st strand cDNA Synthesis kit (Takara Biotechnology Co., Ltd., Dalian, China). Real-time fluorescent quantitative PCR was performed using a SYBR-Green premix (Applied Biosystems, Carlsbad, CA, USA), according to the manufacturer’s instructions, under the following cycling parameters: 1 cycle, 95°C for 7 min; 40 cycles, 95°C for 15 sec and 60°C for 1 min. The following primer sequences were used: Human sterol regulatory element-binding protein-1c sense, 5′-CCACTTCATCAAGGCAGACTCG-3′ and antisense, 5′-CAAGATGGTTCCGCCACTCAC-3′; human FASN sense, 5′-GCTTCCGAGATTCCATCCTACG-3′ and antisense, 5′-GCAGTCAGGCTCACAAACGA-3′; human ACC sense, 5′-GTTATGTGAAAGATGTGGATGA-3′ and antisense, 5′-TGTCTGAAGAGATTAGGGAAGT-3′; human PPAR-α sense, 5′-TCCATCGGCGAGGATAGTTCT-3′ and antisense, 5′-GGTGAAAGCGTGTCCGTGAT-3′; and human CPT-1 sense, 5′-AAATCAATCGGACTCTGGAAACG-3′ and antisense, 5′-TCTTGGTGGCACGACTCACTT-3′.

Cycling conditions were as follows: 95°C for 30 sec, followed by 40 cycles at 95°C for 5 sec and 59°C for 30 sec. Relative PCR product levels were determined based on the threshold cycle value. Glyceraldehyde 3-phosphate dehydrogenase was used as a normalized reference gene. Following amplification, melting curve analysis was completed to verify the accuracy of the amplicon. The 2^−ΔΔCT^ method was used to calculate the relative fold-changes in mRNA expression.

### Western blot analysis

Following CJ treatment, HepG2 hepatocytes were washed with ice-cold PBS and lysed using radioimmunoprecipitation assay buffer with protease and phosphatase inhibitors (Roche Diagnostics, Mannheim, Germany) on ice for 30 min. Insoluble proteins were removed by centrifugation at 14,000 × g at 4°C for 20 min. The cell lysate protein concentration was measured using a bicinchoninic acid protein assay kit (Pierce Biotechnology, Inc., Rockford, IL, USA). Next, 50-μg samples of total protein were loaded and separated by 10% SDS polyacrylamide gel electrophoresis under denaturing and non-reducing conditions. The samples were then transferred to polyvinylidine fluoride membranes (Millipore Corporation, Billerica, MA, USA). The membranes were blocked with 5% non-fat milk in Tris-buffered saline and Tween-20 (TBST) at room temperature for 2 h, which was followed by incubation with a primary antibody at 4°C overnight. Following three TBST washes, the membranes were incubated with secondary antibodies for 1 h at room temperature. Proteins were detected with a chemiluminescence detection system (Pierce Biotechnology, Inc.) and visualized with a LuminoImager (LAS-3000 Bio Imaging analysis system; Fujifilm Co, Ltd., Tokyo, Japan).

### Statistical analysis

All data are expressed as the mean ± SE from three measurements. Statistical significance of the differences among multiple treatment groups was determined through one-way analysis of variance. All the data were analyzed using the SPSS package for Windows (version 18.0; SPSS, Inc., Chicago, IL, USA). P<0.05 was considered to indicate a statistically significant difference.

## Results

### CJ effects on cell viability

**Figure 1:**
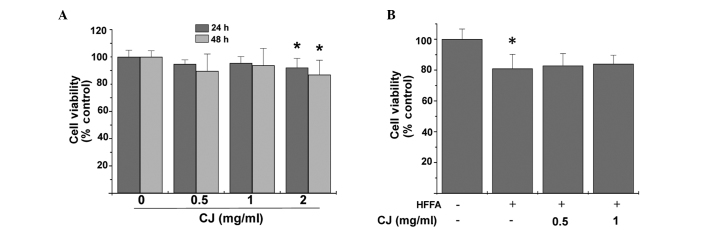
Effects of CJ and HFFA on HepG2 cells. (A) HepG2 cells were incubated with or without CJ at various concentrations and the cell viability was measured using the MTT assay. (B) Effect of HFFA on cytotoxicity in HepG2 cells. Data are expressed as the mean ± SE (bars) of three independent experiments. ^*^P<0.05, vs. control. CJ, *Cirsium japonicum*; HFFA, free fatty acid; MTT, 3-(4,5-dimethylthiazol-2-yl)-2,5-diphenyltetrazolium bromide.

The concentration dependence of the potential cytotoxicity of CJ in HepG2 cells in the absence or presence of 1 mmol/l FFA for 24 h was firstly determined using an MTT assay. CJ exhibited between 1.7 and 12.4% cytotoxicity in HepG2 cells at the 0–2 mg/ml concentration range when incubated for 24 and 48 h, as shown in Fig. 1A. When treated with HFFA for 24 h to induce conditions of hepatic steatosis, 14.3% cytotoxicity was observed in the cells. Nevertheless, no significant cytotoxicity was imparted by CJ, as demonstrated in Fig. 1B.

### CJ decreases lipid accumulation in HFFA-induced steatotic HepG2 cells

**Figure 2:**
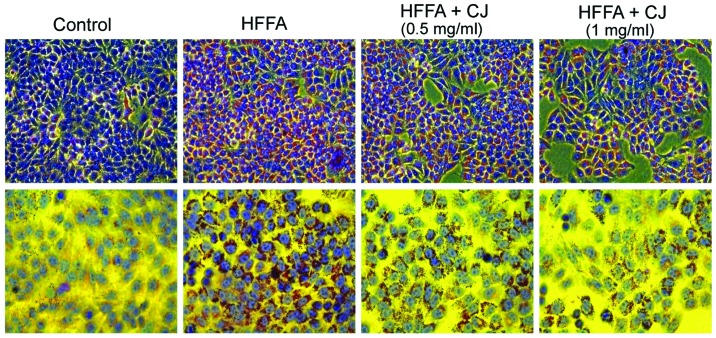
Micrographs of hepatocytes following treatment with 1 mmol/l HFFA and increasing doses of CJ for 24 h. Cells were treated with HFFA alone or with CJ extract for 24 h. Cells were stained with Oil Red O to label the fatty acid accumulation. When CJ was incubated with HFFA, Oil Red O staining was reduced. Upper panel: magnification, ×200; lower panel: magnification, ×400. CJ, *Cirsium japonicum*; HFFA, free fatty acid.

Microscopic examination revealed that HepG2 cells treated with HFFA exhibited significant morphological changes in lipid droplet formation. When CJ was simultaneously administered for 24 h, hepatic lipid accumulation significantly decreased (Fig. 2).

### CJ decreases intracellular TG and TC levels

**Figure 3:**
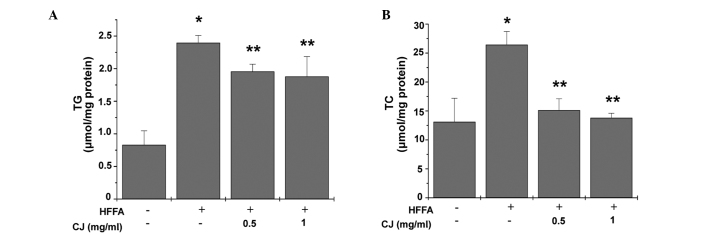
Effect of CJ on (A) TG and (B) TC accumulation in HFFA-induced hepatic steatosis. HFFA-induced accumulation of TG and TC was significantly reduced by CJ cotreatment in HepG2 cells. Vertical bars represent the mean ± SE of three independent experiments. ^*^P<0.05, vs. control; ^**^P<0.05, vs. HFFA group. CJ, *Cirsium japonicum*; HFFA, free fatty acid; TG, triglyceride; TC, total cholesterol.

Following stimulation with FFA for 24 h, intracellular TG and TC levels significantly increased (Fig. 3). However, upon treatment with CJ, TC and TG levels decreased significantly.

### Effect of CJ on the expression levels of genes associated with lipid metabolism

**Figure 4:**
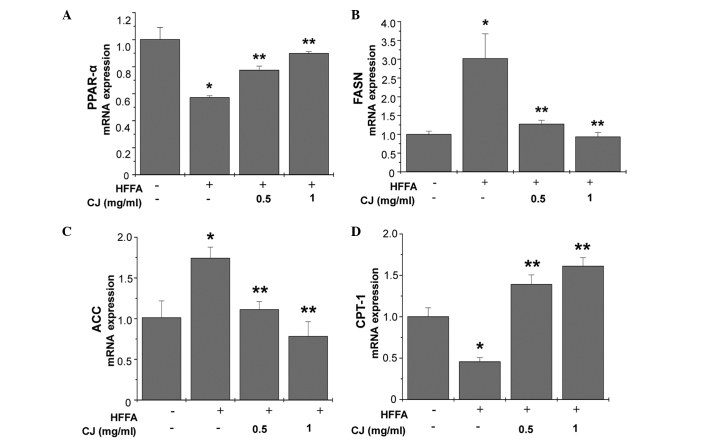
Effects of CJ on lipid metabolism-associated genes in HepG2 cells. Cells were treated with CJ in a concentration-dependent manner for 24 h and (A) PPAR-α, (B) FASN, (C) ACC and (D) CPT-1 mRNA expression levels were determined. HFFA significantly increased the mRNA expression levels of ACC and FASN, while CJ significantly reversed these increases. By contrast, HFFA significantly downregulated the mRNA expression levels of PPAR-α and CPT-1. The effect of CJ cotreatment was a significant reversal and increase in these specific mRNA expression levels in HepG2 cells. Each bar represents the mean ± SE of three independent experiments. ^*^P<0.05, vs. control; ^**^P<0.05, vs. HFFA group. CJ, *Cirsium japonicum*; HFFA, free fatty acid; PPAR-α, peroxisome proliferator-activated receptor-α; CPT-1 carnitine palmitoyltransferase-1; ACC, acetyl-CoA carboxylase; FASN, fatty acid synthase.

Relative mRNA expression levels of lipid metabolism markers were determined using quantitative PCR. FASN and ACC mRNA expression levels were shown to increase relative to β-actin mRNA expression in the HFFA-treated cells (Fig. 4). CJ exhibited an inhibitory effect on the mRNA expression levels of FASN and ACC in the HepG2 cells. By contrast, mRNA expression levels of PPAR-α and CPT-1, regulators of lipolysis and fatty acid transport, were significantly elevated when HepG2 cells were treated with CJ in a concentration-dependent manner.

### CJ inhibits hepatic lipid accumulation via the activation of the AMPK signaling pathway

**Figure 5:**
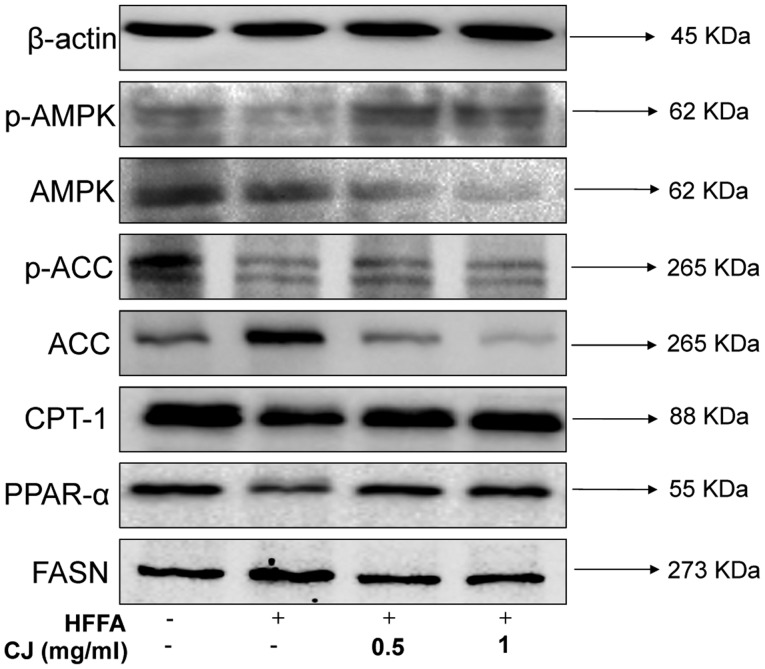
Representative western blot analysis of AMPK and ACC phosphorylation and downstream protein expression levels in HepG2 cells. Cells were cultured with or without 1.00 mmol/l HFFA (OA:PA, 2:1) in the presence or absence of CJ at 0.5 or 1 mg/ml for 24 h. The blots shown are representative of three independent experiments. CJ, *Cirsium japonicum*; HFFA, free fatty acid; AMPK, AMP-activated protein kinase; ACC, acetyl-CoA carboxylase; CPT-1, carnitine palmitoyltransferase-1; PPAR-α, proliferator-activated receptor-α; FASN, fatty acid synthase; OA, oleic acid; PA, palmitic acid.

To investigate whether CJ-mediated lipid reduction proceeded via AMPK signaling, the Thr172-phosphorylation status of AMPK, an essential marker of AMPK activity, was determined. As shown in Fig. 5, CJ treatment significantly increased AMPK phosphorylation, as well as the phosphorylation of its direct substrate, ACC, in a concentration-dependent manner. To determine whether AMPK activation affected the downstream target genes, the products of ACC activity were investigated due to their essential role in the regulation of fatty acid metabolism. Consistent with the gene expression profiles, CJ was shown to increase the protein expression levels of CPT-1 and PPAR-α, which are associated with fatty acid oxidation. In addition, CJ-facilitated downregulation of FASN, one of the key enzymes involved in lipid synthesis, was observed, which can catalyze acetyl coenzyme A and malonyl coenzyme A synthesis of TGs.

## Discussion

NAFLD is the most common symptomless liver disease worldwide, characterized by steatosis or fat accumulation in the liver (14). It is estimated that the prevalence of NAFLD in the general USA population is ~20% with anticipated annual increases (15). Despite the high prevalence of NAFLD and its potential for serious liver damage, currently available therapies based on the modifications of diet and physical activity (16) may be insufficient to address the disease in all patients. Natural products, including traditional Chinese medicines (TCMs), have been applied clinically for the treatment of various metabolic syndromes and diseases, including NAFLD. The general effects of such therapies are hypothesized to result from the broad targeting of multiple molecular pathways. In addition, TCMs have relatively few adverse side effects. In this category of herbal therapies, CJ is a traditional Chinese perennial herb with a number of known pharmacological applications. The main constituents of CJ include flavonoids, essential oils, triterpene and sterol. Although CJ has a long history of use in TCM, the molecular mechanisms underlying the biological effects have been understudied.

AMPK, an important metabolic regulator, functions as a ‘master switch’ by phosphorylating specific target enzymes (17). Activation of AMPK in the liver results in the stimulation of fatty acid oxidation and the inhibition of lipogenesis, glucose production and protein synthesis. In addition, liver AMPK activation causes the direct phosphorylation and inactivation of ACC, which leads to decreased conversion of acetyl-CoA to malonyl-CoA and downregulated fatty acid synthesis (18). Alternatively, AMPK positively regulates fatty acid oxidation by activating PPAR-α and PPAR-γ coactivator-1 (19). In light of these effects, identifying pharmacological agents that stimulate AMPK activity in hepatocytes may provide effective treatment options for fatty liver disease.

A number of herbal medicines, including resveratrol, *Artemisia scoparia* and berberine, have demonstrated efficacy in treating and preventing obesity and associated metabolic disorders via the regulation of AMPK activity (20–22). Considering the key role of AMPK activation in regulating lipid metabolism, we hypothesized that AMPK may play a vital role in mediating the effects of CJ against hepatic lipid accumulation.

In the present study, HepG2 cells were supplemented with pathophysiological levels of HFFA to mimic the influx of excess FFAs in hepatocytes, with the result of hepatic steatosis. Upon establishing this successful hepatic steatosis model, the observations demonstrated that exposure of HepG2 cells to HFFA increased the levels of intracellular TG and TC. Addition of CJ decreased HFFA-stimulated hepatic TG and TC accumulation. To gain a better understanding of the potential molecular mechanisms underlying the effects of CJ in the *in vitro* model, the influence of CJ on AMPK activation and the associated genes in fatty acid metabolism was investigated. CJ triggered an increase in the phosphorylation of AMPK and its direct substrate, ACC, indicating that CJ activated AMPK. Furthermore, CJ treatment modulated the expression levels of genes associated with lipid metabolism, including FASN, PPAR-α and CPT-1. To the best of our knowledge, the results of the present study are the first to hypothesize that CJ may serve as a therapeutic tool to attenuate hepatic steatosis by targeting the hepatic AMPK system.

In conclusion, the present study demonstrated that CJ treatment reduced the levels of TC and TG in steatotic HepG2 cells and promoted the phosphorylation of AMPK and ACC. From these results, we hypothesize that a major mechanism underlying the beneficial effects of CJ in reducing hepatic lipid accumulation is via activating AMPK. Thus, CJ possesses great potential for preventing NAFLD and nonalcoholic steatohepatitis. Future research is likely to provide further insight into the benefits and mechanisms of CJ in the treatment of NAFLD and other liver diseases.

## References

[1] Koo SH (2013). Nonalcoholic fatty liver disease: molecular mechanisms for the hepatic steatosis. Clin Mol Hepatol.

[2] Gentile CL, Pagliassotti MJ (2008). The role of fatty acids in the development and progression of nonalcoholic fatty liver disease. J Nutr Biochem.

[3] Kaila B, Raman M (2008). Obesity: a review of pathogenesis and management strategies. Can J Gastroenterol.

[4] Angulo P (2007). Obesity and nonalcoholic fatty liver disease. Nutr Rev.

[5] Marra F, Gastaldelli A, Svegliati Baroni G, Tell G, Tiribelli C (2008). Molecular basis and mechanisms of progression of non-alcoholic steatohepatitis. Trends Mol Med.

[6] Parekh S, Anania FA (2007). Abnormal lipid and glucose metabolism in obesity: implications for nonalcoholic fatty liver disease. Gastroenterology.

[7] Gómez-Lechón MJ, Donato MT, Martínez-Romero A, Jiménez N, Castell JV, O’Connor JE (2007). A human hepatocellular in vitro model to investigate steatosis. Chem Biol Interact.

[8] Ricchi M, Odoardi MR, Carulli L (2009). Differential effect of oleic and palmitic acid on lipid accumulation and apoptosis in cultured hepatocytes. J Gastroenterol Hepatol.

[9] Stapleton D, Mitchelhill KI, Gao G (1996). Mammalian AMP-activated protein kinase subfamily. J Biol Chem.

[10] Misra P (2008). AMP activated protein kinase: a next generation target for total metabolic control. Expert Opin Ther Targets.

[11] Viollet B, Mounier R, Leclerc J, Yazigi A, Foretz M, Andreelli F (2007). Targeting AMP-activated protein kinase as a novel therapeutic approach for the treatment of metabolic disorders. Diabetes Metab.

[12] Saha AK, Avilucea PR, Ye JM, Assifi MM, Kraegen EW, Ruderman NB (2004). Pioglitazone treatment activates AMP-activated protein kinase in rat liver and adipose tissue in vivo. Biochem Biophys Res Commun.

[13] Park JC, Hur JM, Park JG, Kim SC, Park JR, Choi SH, Choi JW (2004). Effects of methanol extract of Cirsium japonicum var. ussuriense and its principle, hispidulin-7-O-neohesperidoside on hepatic alcohol-metabolizing enzymes and lipid peroxidation in ethanol-treated rats. Phytother Res.

[14] Clark JM, Brancati FL, Diehl AM (2002). Nonalcoholic fatty liver disease. Gastroenterology.

[15] Ruhl CE, Everhart JE (2004). Epidemiology of nonalcoholic fatty liver. Clin Liver Dis.

[16] Ha SK, Kim J, Chae C (2011). Role of AMP-activated protein kinase and adiponectin during development of hepatic steatosis in high-fat diet-induced obesity in rats. J Comp Pathol.

[17] Federico A, Niosi M, Vecchio Blanco CD, Loguercio C (2008). Emerging drugs for nonalcoholic fatty liver disease. Expert Opin Emerg Drugs.

[18] Viollet B, Foretz M, Guigas B (2006). Activation of AMP-activated protein kinase in the liver: a new strategy for the management of metabolic hepatic disorders. J Physiol.

[19] Lee WJ, Kim M, Park HS (2006). AMPK activation increases fatty acid oxidation in skeletal muscle by activating PPARalpha and PGC-1. Biochem Biophys Res Commun.

[20] Lagouge M, Argmann C, Gerhart-Hines Z (2006). Resveratrol improves mitochondrial function and protects against metabolic disease by activating SIRT1 and PGC-1alpha. Cell.

[21] Wang ZQ, Zhang XH, Yu Y (2013). *Artemisia scoparia* extract attenuates non-alcoholic fatty liver disease in diet-induced obesity mice by enhancing hepatic insulin and AMPK signaling independently of FGF21 pathway. Metabolism.

[22] Quan HY, Kim do Y, Kim SJ, Jo HK, Kim GW, Chung SH (2013). Betulinic acid alleviates non-alcoholic fatty liver by inhibiting SREBP1 activity via the AMPK-mTOR-SREBP signaling pathway. Biochem Pharmacol.

